# Stimulation of muscle protein synthesis with low-dose amino acid composition in older individuals

**DOI:** 10.3389/fnut.2024.1360312

**Published:** 2024-03-08

**Authors:** David D. Church, Arny A. Ferrando, Robert R. Wolfe

**Affiliations:** Department of Geriatrics, Donald W. Reynolds Institute on Aging, Center for Translational Research in Aging and Longevity, University of Arkansas for Medical Sciences, Little Rock, AR, United States

**Keywords:** essential amino acids (EAAs), muscle protein synthesis, anabolism, aging, stable isotope tracer

## Abstract

Essential amino acid (EAA)-based compositions have been shown to be effective stimulators of muscle protein synthesis, but the lower limit of effective dosage is not clear. We have used stable isotope tracer methodology to quantify the response of muscle protein fractional synthetic rate (FSR) to a dose of 3.6 g of a high-leucine composition of EAAs plus arginine in older subjects. Muscle protein FSR increased 0.058%/hour over 3 h following consumption. When account was taken of the total muscle mass, this increase in muscle protein FSR represented approximately 80% of ingested EAAs. We conclude that a low dose of an EAA-based composition can effectively stimulate muscle protein synthesis.

## Introduction

Essential amino acid (EAA)-based nutritional supplements have become popular for increasing muscle mass and physical function [e.g., (1)]. Two important attributes of free-form EAA-based nutritional supplements are that the specific composition can be configured to target a variety of conditions and circumstances, and that free-form EAAs require no digestion and are absorbed rapidly and completely. The complete and rapid absorption of free EAAs enables small doses to reach higher peak concentrations and speed to achieving peak concentrations than intact dietary protein supplements, meaning that consumption of free-form EAA compositions can potentially act as metabolic regulators. In contrast, intact dietary proteins have slower rates of digestion and absorption of EAAs, with the result that plasma EAA concentrations rarely rise sufficiently to serve as metabolic signals. The stimulation of muscle protein synthesis by free EAAs is of particular interest, as muscle protein synthesis is the metabolic basis for increased muscle mass and function (2). Free EAA compositions have been shown to stimulate muscle protein synthesis more than the same profile and amount of EAAs in dietary protein (3), and EAA profiles designed for specific applications may provide even greater advantages than the profile of EAAs in high-quality proteins on a per gram basis (4).

Essential amino acid-based compositions containing a high proportion of leucine have become appreciated for the stimulation of muscle protein synthesis, which is the metabolic basis for increased muscle mass and physical function (2). High-leucine compositions have proven to be particularly useful for stimulating muscle protein fractional synthesis rate (FSR) and physical function in older individuals who may have some degree of anabolic resistance, meaning a diminished stimulatory effect of dietary protein on muscle protein synthesis (4, 5). Most studies testing the effect of EAA-based compositions on muscle protein FSR have used 6 g or more of active product. However, a smaller dose would have advantages in terms of convenience, taste, and cost. We recently collated the data from several studies in which increases in plasma EAA concentrations were related to the extent of stimulation of muscle protein synthesis (6). The slope of the relation between increase in EAA concentration and the stimulation of muscle protein synthesis intersected the *x*-axis at a muscle fractional synthetic rate (FSR) at a value greater than 0. This predicts that even a very small increase in plasma EAA concentration, consistent with ingestion of as little as 1–4 g EAAs, will stimulate muscle FSR. However, results of muscle protein FSR after consumption of small amounts of EAAs are equivocal. For example, consumption of 1.5 g of EAAs induced a transient increase in muscle protein FSR, but not when the response was evaluated over a longer time interval (7). Consequently, in the current study, we have quantified the response of muscle protein FSR to consumption of 3.6 g of a high-leucine EAA-based composition designed specifically for older individuals. The response of muscle protein FSR was measured over a 3-h time interval, considered the minimal time frame over which to evaluate physiologically relevant changes (1).

## Methods

### Study design

Twelve healthy subjects participated in this study. Subject characteristics are shown in Table 1.

**Table 1:** Subject characteristics.

	Male	Female	Total
Age (y)	69 ± 3	68 ± 3	69 ± 2
Sex	6	6	12
Body weight (kg)	98.2 ± 5.3	71.7 ± 6.3	83.7 ± 5.8
Height (cm)	183.8 ± 4.1	164.6 ± 1.7	173.3 ± 3.6
BMI (kg/m^2^)	29.0 ± 1.2	26.3 ± 1.9	27.6 ± 1.2

The study was approved by the Institutional Review Board of The University of Arkansas for Medical Sciences. Muscle protein FSR was measured by means of the primed constant infusion of L-[ring-^2^H_5_] phenylalanine, as described previously (8). The tracer was infused for a total of 8 h. Muscle protein FSR was quantified in the post-absorptive state over hours 3–5 of tracer infusion, after which 3.6 g of an EAA-based beverage was consumed. Post-prandial FSR was measured between hours 5 and 8 of tracer infusion. Muscle biopsies were taken at 3, 5, and 8 h after initiation of tracer infusion to determine muscle protein FSR. Muscle protein FSR was calculated as the rate of tracer incorporation divided by the precursor enrichment, taken to be the average intracellular phenylalanine enrichment obtained from the biopsy samples (8). Blood samples were obtained at the end of the post-absorptive state and throughout the post-prandial state for measurement of EAA concentrations. EAA concentration values are expressed as area under the curve (AUC) of the increase in EAAs over the 3-h post-prandial period.

### Study product

The EAA-based composition was a proprietary blend containing 1.34 g leucine, 0.558 g lysine, 0.367 g valine, 0.355 g isoleucine, 0.330 g arginine, 0.288 g threonine, 0.224 g phenylalanine, 0.110 g methionine, 0.055g histidine, and 0.002 g tryptophan. The blend of EAAs (especially the high proportion of leucine) targeted the stimulation of muscle protein synthesis in older individuals (4). Arginine was included even though it can be produced metabolically in the bod because it is a “conditionally” essential amino acid (9). In many circumstances, including aging, endogenous arginine production may not provide an adequate amount (9). In addition, arginine increases the stimulation of muscle protein synthesis by an EAA composition (10). The powder was dissolved in approximately 200 mL water for consumption. The product is commercially available under a variety of names including Rejinator, Muscle Rescue, Rejuvenate, and XymolX.

### Statistical analysis

Data are presented as means ± SEM. A group × time repeated measures ANOVA was used to analyze muscle FSR changes with statistical significance designated at α ≤ 0.05.

## Results

**Figure 1:**
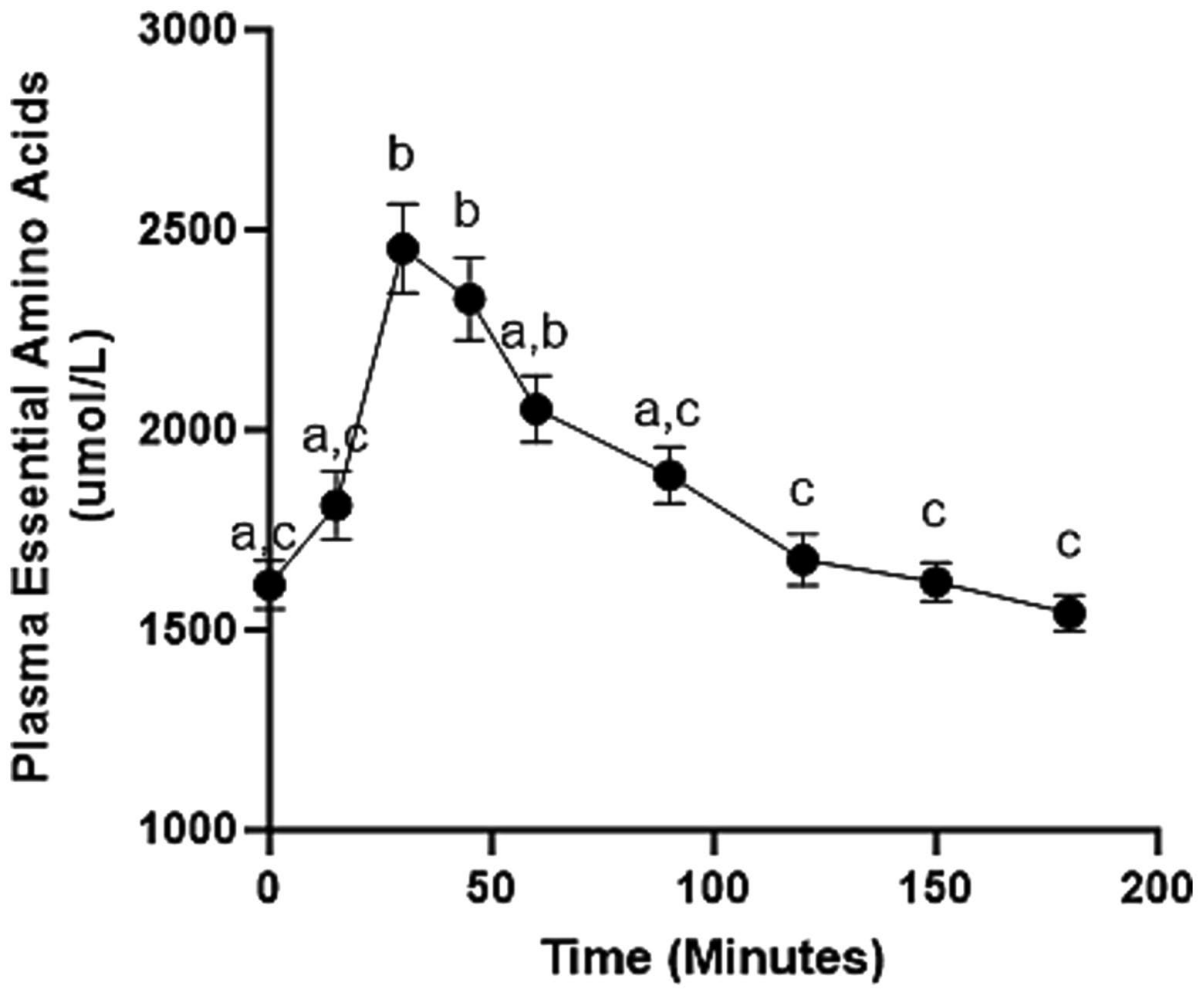
Plasma essential amino acid concentrations following ingestion of 3.6 g of crystalline essential amino acids at time 0. Values are mean ± SEM. Times not sharing the same letter are significantly different (*p* ≤ 0.05) from each other.

A significant main effect of time (*p* ≤ 0.001) was observed for the increase in muscle FSR (0.0568 ± 0.0338%/h) from the post-absorptive state to the post-prandial state. This response represented a 48.9% increase in muscle protein FSR over the basal value. The total EAA concentration response (AUC) was 185.4 mol/L/min over the post-prandial value (Figure 1).

## Discussion

The principal result of this study is that consumption of 3.6 g of an EAA-based composition significantly stimulated muscle protein FSR in healthy older subjects. The stimulation of muscle protein FSR is the basis for improved muscle mass and function, and therefore it is reasonable to expect that regular consumption of 3.6 g of the EAA-based composition would result in beneficial physiological effects. Further, rough calculations of how much of the EAA-based consumed composition was converted into muscle protein indicates that the composition was highly effective. If we assume that the average muscle mass is approximately 30 kg, of which 20% is protein (11), the total amount of skeletal muscle protein produced over 3 h can be estimated to be 10.3 g (0.00058%/h x 3 h x 30,000 g muscle mass x 0.2 g skeletal muscle protein/g muscle mass = 10.4 g of skeletal muscle protein). This estimation is dependent on an assumed muscle mass of 30 kg, but even if we assume a skeletal muscle mass of half that value the amount of protein produced is still greater than the amount of the EAA-based composition consumed (0.00058%/h x 3 h x 15,000 g muscle mass x 0.2 g protein/g muscle mass = 5.3 g protein produced vs. 3.6 g EAA-based composition consumed). The greater amount of skeletal muscle produced than amino acids consumed can be explained in part by incorporation of endogenous non-essential amino acids into newly synthesized muscle protein. Human skeletal muscle is approximately 40% EAAs (12), so for any amount of EAAs incorporated into newly synthesized muscle protein approximately 1.5 times that amount of endogenous non-essential amino acids are also required to produce muscle protein. When the contribution to muscle protein synthesis of the exogenous EAAs is distinguished from the contribution of endogenous non-essential amino acids, it can be estimated that approximately 60–110% of ingested EAAs were incorporated into newly synthesized muscle protein for assumed muscle masses of 15 and 30 kg, respectively ([5.3 g protein produced x 0.4 g EAA incorporated into muscle protein per g protein produced] x 100%/3.6 g consumed for a muscle mass of 15 kg and [10.4 g protein produced x 0.4 g EAA incorporated into muscle protein per g protein produced] x 100%/3.6 g consumed for a muscle mass of 30 kg). We can consider these calculated values as upper and lower bounds of the likely amount of consumed EAAs incorporated into muscle protein, meaning that it is reasonable to assume that approximately 80% of ingested EAAs were incorporated into muscle protein.

**Figure 2:**
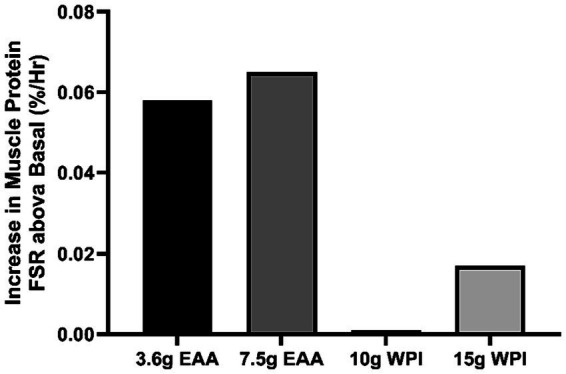
Increase above basal value in muscle protein fractional synthetic (FSR) rate after consumption of 3.6 g of essential amino acids (EAA) from this investigation, 7.5 g of EAA (Reference 13), 10 g of whey protein isolate (WPI; Reference 14), and 15 g of WPI (Reference 3).

Our estimation that approximately 80% of the 3.6 g dose of ingested EAAs was incorporated into muscle protein indicates a high anabolic efficiency, even in older individuals who may have decreased anabolic responsiveness to EAAs (4). The high anabolic efficiency of the EAA-based composition may have been in part due to the small dose consumed. According to the same calculations described above, the lower and upper bounds of the percent of exogenous EAAs incorporated into muscle protein were 30–60% of a dose of 7.5 g of an EAA composition similar to the one used in the current study in older subjects that closely matched the subjects in our study (13). The response we observed to 3.6 g of the EAA-based composition was greater than when the same amount or more EAAs, along with non-essential amino acids, were consumed in the format of dietary protein. For example, consumption of 10 g of whey protein isolate, containing approximately 4 g of EAA, failed to elicit a significant stimulation of muscle FSR in young, healthy subjects at rest (14), and consumption of 15 g whey protein isolate containing approximately 6 g of EAAs increased muscle protein FSR only 0.017%/h in older subjects at rest (3). The responses to different doses of EAAs and whey protein are shown in Figure 2.

Several factors could have contributed to the anabolic efficiency of the EAA-based composition we used in this study. The free-form EAAs require no digestion and are completely and rapidly absorbed, while the efficiency of digestion of whey protein is less than 100% and may be impeded in elderly (15). The more complete and rapid absorption of free EAAs is associated with a more rapid and greater total increase in plasma EAA concentrations than when a dietary protein is consumed, and this difference may have contributed to the greater anabolic response to the EAA-based composition (6). In addition, the formulation of the EAA-based composition was based on maximizing the muscle protein synthetic response, and although whey protein contains as many or more total EAAs, the profiles of the EAAs in whey protein differed significantly from the profile in the EAA-based composition. Further, the metabolic disposition of absorbed EAAs likely depends on the dosage. In addition to incorporation into muscle protein, dietary EAAs can potentially be incorporated into other proteins in the body and can be oxidized as well. However, it is likely that in our study these other fates of the ingested EAAs were minimal. The highly anabolic response to the low-dose EAAs would be expected to minimize the fraction of EAAs that were oxidized (16). Further, it is likely that muscle protein is more sensitive to the anabolic signal of an increase in EAA concentrations than proteins in other tissues and organs (17). The increase in plasma EAA concentrations following consumption of the 3.6 g dose of the EAA-based composition was modest and may have been insufficient to stimulate protein FSR in tissues and organs other than muscle. The other tissues and organs may play an increasingly important role in the disposition of absorbed EAAs as the amount of EAAs consumed increases.

## Limitations and conclusions

Although no control group was provided, previous reports have shown oral EAA to increase muscle FSR above basal values. Further, reports have demonstrated the effect greater than isocaloric and iso-EAA whey protein bolus (7). Further, previous research has demonstrated the increase in muscle FSR following the ingestion of protein is due to the EAA component of a protein source (1, 6). Thus, our data indicate a 3.6 g dose of an EAA-based nutritional composition was highly efficient in stimulating muscle protein synthesis.

## Data availability statement

Data presented in the article will be made available upon request pending application to and approval from the corresponding author.

## Ethics statement

The studies involving humans were approved by University or Arkansas for Medical Science IRB HRAC#: 89220. The studies were conducted in accordance with the local legislation and institutional requirements. The participants provided their written informed consent to participate in this study.

## Author contributions

DC: Data curation, Formal analysis, Writing – original draft, Writing – review & editing. AF: Conceptualization, Methodology, Project administration, Resources, Supervision, Writing – original draft, Writing – review & editing. RW: Conceptualization, Data curation, Funding acquisition, Investigation, Methodology, Project administration, Resources, Supervision, Writing – original draft, Writing – review & editing.
