# Erratum: Stimulation of muscle protein synthesis with low-dose amino acid composition in older individuals

**DOI:** 10.3389/fnut.2024.1415503

**Published:** 2024-04-22

**Authors:** Frontiers Production Office

**Affiliations:** Frontiers Media SA, Lausanne, Switzerland

**Keywords:** essential amino acids (EAAs), muscle protein synthesis, anabolism, aging, stable isotope tracer

Due to a production error, a mistake was made in **Methods**, subsection Study product. The sentence reads “The EAA-based composition was a proprietary blend containing 1.34 g leucine, 0.558 g lysine, 0.367 g valine, 0.355 g isoleucine, 0.330 g arginine, 0.288 g threonine, 0.288 g phenylalanine, 0.110 g methionine, 0.055g histidine, and 0.055 g tryptophan.”

A correction has been made and the sentence should read as follows: “The EAA-based composition was a proprietary blend containing 1.34 g leucine, 0.558 g lysine, 0.367 g valine, 0.355 g isoleucine, 0.330 g arginine, 0.288 g threonine, 0.224 g phenylalanine, 0.110 g methionine, 0.055g histidine, and 0.002 g tryptophan.”

The publisher apologizes for this mistake. The original article has been updated.

